# Response to Emergence of Middle East Respiratory Syndrome Coronavirus, Abu Dhabi, United Arab Emirates, 2013–2014

**DOI:** 10.3201/eid2207.160040

**Published:** 2016-07

**Authors:** Farida Ismail Al Hosani, Kimberly Pringle, Mariam Al Mulla, Lindsay Kim, Huong Pham, Negar N. Alami, Ahmed Khudhair, Aron J. Hall, Bashir Aden, Feda El Saleh, Wafa Al Dhaheri, Zyad Al Bandar, Sudhir Bunga, Kheir Abou Elkheir, Ying Tao, Jennifer C. Hunter, Duc Nguyen, Andrew Turner, Krishna Pradeep, Jurgen Sasse, Stefan Weber, Suxiang Tong, Brett L. Whitaker, Lia M. Haynes, Aaron Curns, Susan I. Gerber

**Affiliations:** Health Authority Abu Dhabi, Abu Dhabi, United Arab Emirates (F.I. Al Hosani, M. Al Mulla, A. Khudhair, B. Aden, F. El Saleh, W. Al Dhaheri, Z. Al Bandar, K. Abou Elkheir, A. Turner, K. Pradeep);; Centers for Disease Control and Prevention, Atlanta, Georgia, USA (K. Pringle, L. Kim, H. Pham, N.N. Alami, A.J. Hall, S. Bunga, Y. Tao, J.C. Hunter, D. Nguyen, S. Tong, B.L. Whitaker, L.M. Haynes, A. Curns, S.I. Gerber);; Sheikh Khalifa Medical Laboratory, Abu Dhabi (J. Sasse, S. Weber).

**Keywords:** Middle East respiratory syndrome coronavirus, asymptomatic infection, risk factors, MERS-CoV, United Arab Emirates, public health surveillance, clusters, respiratory disease, viruses

## Abstract

In January 2013, several months after Middle East respiratory syndrome coronavirus (MERS-CoV) was first identified in Saudi Arabia, Abu Dhabi, United Arab Emirates, began surveillance for MERS-CoV. We analyzed medical chart and laboratory data collected by the Health Authority–Abu Dhabi during January 2013–May 2014. Using real-time reverse transcription PCR, we tested respiratory tract samples for MERS-CoV and identified 65 case-patients. Of these patients, 23 (35%) were asymptomatic at the time of testing, and 4 (6%) showed positive test results for >3 weeks (1 had severe symptoms and 3 had mild symptoms). We also identified 6 clusters of MERS-CoV cases. This report highlights the potential for virus shedding by mildly ill and asymptomatic case-patients. These findings will be useful for MERS-CoV management and infection prevention strategies.

## Abstract 2

We found that this virus may be detected in mildly ill and asymptomatic case-patients.

Middle East respiratory syndrome coronavirus (MERS-CoV) was first identified in October 2012 in Saudi Arabia (*1*). By November 6, 2015, the World Health Organization (WHO) had received reports of 1,611 cases and at least 575 deaths caused by MERS-CoV. Index cases that occurred outside the Arabian Peninsula all had a link to the region, such as recent travel (*2*–*11*). Most contact investigations outside the Arabian Peninsula have identified limited spread to additional persons (*5*,*6*,*10*), with the exception of the recent South Korea outbreak, in which 186 persons were infected (*11*). Within the Arabian Peninsula, clusters have been identified among extended families, households, and healthcare settings (*12*,*13*).

Abu Dhabi is the largest emirate in the United Arab Emirates (UAE), with a diverse and mostly expatriate population of 2.3 million persons. The Health Authority–Abu Dhabi (HAAD) is the healthcare sector regulatory body and the custodian of public health in the emirate. In this role, HAAD directs the public health response for MERS-CoV, in conjunction with the UAE Ministry of Health. The Emirate of Abu Dhabi has 3 regions: the Western Region, which borders Saudi Arabia; Abu Dhabi, which includes a large, mostly urban population; and the Eastern Region, which borders Oman.

Given the emirate’s proximity to Saudi Arabia, HAAD created a standardized public health response protocol for MERS-CoV in January 2013 with the following objectives: 1) educate physicians by circulating regular instructions to all healthcare facilities, which contained WHO definitions for confirmed and probable cases and the mechanism for handling suspected cases and reporting to HAAD; 2) ensure laboratory capacity to test for MERS-CoV; and 3) add reporting options for MERS-CoV to existing electronic surveillance systems or create new surveillance systems to detect MERS-CoV. This article describes the public health response to the emergence of MERS-CoV in the region and epidemiologic characteristics of patients with laboratory-confirmed MERS-CoV infection in Abu Dhabi during January 1, 2013–May 9, 2014.

## Methods

### Definitions

A case was defined as laboratory-confirmed MERS-CoV infection in a person as determined by a PCR test using 2 gene targets (*14*). Cases were further classified into epidemiologic categories. A household case was illness in a person who spent >1 night or 8 continuous hours in the same home with a case-patient while that person was infected. A healthcare-associated case (HCA) was illness in a case-patient who had been exposed to another case-patient exclusively in a healthcare setting in the 14 days before their own onset of symptoms or specimen collection date. A work (or other) setting–related case was illness in a person who was exposed to a case-patient but not in a healthcare or household setting. An epidemiologically unlinked case was defined as illness in a person without any documented link to a patient with suspected or confirmed MERS-CoV infection. Contacts were persons who provided care for the patient, including healthcare personnel or family members; had close physical contact with the patient; or stayed in the same place with a laboratory-confirmed.

A household cluster was defined as >2 cases in the same household. An HCA cluster was >2 HCA cases in the same healthcare setting or healthcare interaction (e.g., hospital transport). A work cluster was >2 cases in the same workplace. To be considered part of the same cluster, secondary case-patients must have had a positive laboratory test result and share an epidemiologic link, such as a workplace, hospital room, or household. Suspected case-patients who had a negative laboratory test result for MERS-CoV were classified as test-negative suspected case-patients.

Asymptomatic case-patients were those who had no reported symptoms at the time of a positive test recorded by a healthcare provider in the medical chart. Mildly symptomatic case-patients reported symptoms, such as sore throat, rhinorrhea, or cough, and did not require oxygen during their hospital stay. Severely symptomatic case-patients required supplemental oxygenation during their hospitalization, ranging from nasal cannula to intubation.

### Surveillance System 

HAAD began surveillance for MERS-CoV in January 2013. Three surveillance systems were used to identify case-patients who tested positive for the virus: 1) Infectious Disease Electronic Notification System, in which physicians identified suspected case-patients and completed a basic form on the basis of patients’ clinical features; 2) Sheikh Khalifa Medical Center Laboratory surveillance, at the laboratory responsible for processing MERS-CoV specimens, which also records patient demographic characteristics, dates of collection, and results; and 3) Operations Center, which runs an active surveillance system that contacts 42 public and private hospitals in Abu Dhabi and records information regarding patients that were admitted or transferred to the intensive care unit because of primary respiratory failure or acute respiratory distress syndrome. 

Because suspected case-patients may be admitted for primary respiratory failure or acute respiratory distress syndrome from multiple etiologies, HAAD authorities conducted investigations to determine whether an identified patient should be tested for MERS-CoV, if the patient had not already been captured by 1 of the surveillance systems. In addition, during April and May 2014, screenings for MERS-CoV were conducted, most under the supervision of HAAD, in various locations where MERS-CoV cases had been identified, including, but not limited to, hospitals and workplace settings.

For each case-patient, HAAD conducted detailed contact investigations within 24 hours of notification. HAAD officials interviewed case-patients if available. If the case-patient was unable to be interviewed, the patient’s epidemiologic information was collected from family members or other proxies. Staff at hospitals or local Disease and Prevention Screening Centers tested a sputum (preferred) or nasopharyngeal sample from all case-patient contacts for MERS-CoV. Samples from contacts were tested regardless of whether the person experienced symptoms. For each test-positive case-patient identified, we collected clinical information using the International Severe Acute Respiratory and Emerging Infection Consortium form, which was filled out in real time by healthcare providers and later verified by retrospective chart review.

All persons who tested positive for MERS-CoV, including asymptomatic persons, were admitted to a hospital. Before they could be discharged, confirmed case-patients were required to show negative results on 2 MERS-CoV PCR tests conducted at least 48 hours apart.

### Laboratory Diagnostic Testing

The laboratory analyzed upper (e.g., nasopharyngeal, oropharyngeal) and lower respiratory tract samples (e.g., sputum, bronchoalveolar lavage fluid, tracheal aspirates) and serum. If hospital staff were not able to collect sputum spontaneously, the clinician could order that sputum be induced in a negative pressure room or that nasopharyngeal aspirates be obtained. Specimens were tested by using real-time reverse transcription PCR (rRT-PCR), upE, and open reading frame 1 assays in the Sheikh Khalifa Medical Center laboratory (*15*,*16*). A laboratory team from the US Centers for Disease Control and Prevention (CDC; Atlanta, GA, USA) used a nucleocapsid-based rRT-PCR assay to verify infection in a random sample of 23 specimens from 2014 (*17*). To calculate the length of positivity for each MERS-CoV case-patient, we calculated the difference between the date of the first positive test and the date the virus was last detected.

### Data Management and Analysis

We merged results from Sheikh Khalifa Medical Center laboratory and HAAD’s epidemiologic databases, which contained information on patients’ demographic characteristics, symptoms, and exposure, using a combination of unique individual identifiers. We also retrospectively reviewed medical charts to collect additional clinical information about case-patients with positive test results.

Because changes occurred in the surveillance system during the study period, we compared demographic characteristics of MERS-CoV case-patients and those of suspected case-patients who were tested and had negative results during January 1, 2013–April 17, 2014. Suspected case-patients with negative test results identified during April 18, 2014–May 9, 2014, were not available for comparison. We included all MERS-CoV case-patients identified during January 1, 2013–May 9, 2014, in analyses of demographic characteristics, clusters, disease severity, sample type, and PCR positivity. Differences in proportions were contrasted by using Mantel-Haenszel χ^2^ test. We defined statistical significance as a p value <0.05. Data were analyzed by using SAS version 9.3 (SAS Institute, Cary, NC, USA).

### Ethics

Because these data were collected as part of a public health response, HAAD and CDC determined that data were nonresearch and not subject to review by an institutional review board. Secondary data analysis was then carried out for operational purposes.

## Results

### Comparison of Laboratory-Confirmed Case-Patients and Test-Negative Suspected Case-Patients

For January 1, 2013–April 17, 2014, HAAD surveillance systems contained records for 1,586 unique persons, including 41 (3%) with confirmed MERS-CoV infection, 1,467 (92%) suspected case-patients with negative test results, and 78 (5%) whose test results were unknown or no test had been performed. Most case-patients were male (61%), Asian (54%), and 20–59 years of age (76%) (Technical Appendix Table 1). Case-patients more frequently reported exposure to a known or suspected MERS-CoV case-patient (30, 73%) and exposure to an animal (10, 24%) within the previous 2 weeks of illness onset or specimen collection than did suspected case-patients with negative test results. Fifty-one percent of MERS-CoV cases were healthcare associated.

Emiratis and nationals from other Gulf Cooperation Council countries (i.e., Saudi Arabia, United Arab Emirates, Qatar, Bahrain, and Oman) were the groups most often tested (855, 57%), whereas Asians had the highest proportion of positive test results (22/187, 12%). Of all reported and evaluated case-patients, 15% (6/41) had traveled internationally (Technical Appendix Table 1).

### All MERS-CoV Cases

During April 17–May 9, 2014, surveillance activities identified an additional 24 MERS-CoV cases, for a total of 65 cases during the January 1, 2013–April 17, 2014, study period. We retrospectively reviewed charts of 64/65 (98%) case-patients; 1 case-patient was transferred to a hospital outside Abu Dhabi, and medical records were unavailable.

The first case-patient with confirmed MERS-CoV infection in Abu Dhabi was identified in March 2013, and the number of case-patients identified (n = 41) peaked in April 2014 (Figure). During April 2014, the highest number of tests was performed, with 24 positive results, 323 negative results, and no test result available for 64 tests. Through contact tracing, HAAD identified 2,372 contacts for 56 case-patients, an average of 56 contacts (range 2–199 contacts) per positive case-patient.

**Figure:**
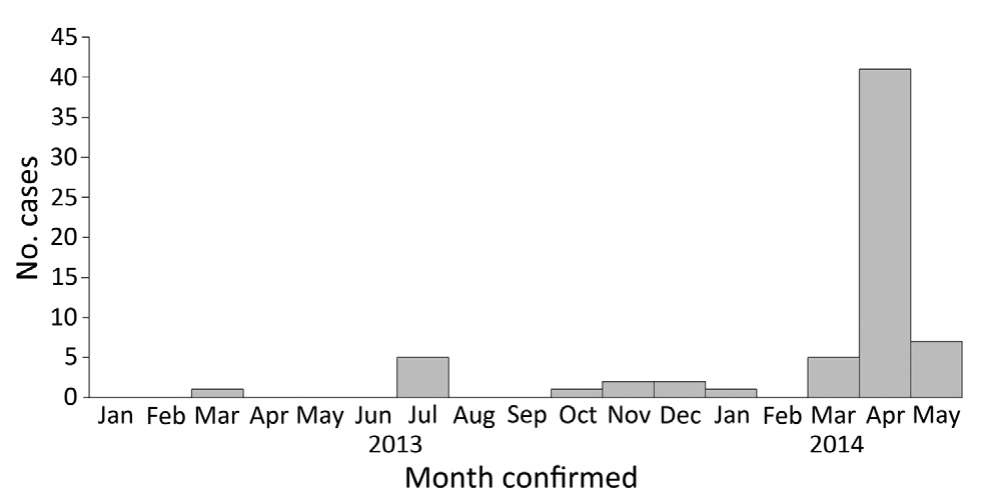
Epidemiologic curve showing confirmed cases of Middle East respiratory syndrome coronavirus (MERS-CoV) infection, Abu Dhabi, United Arab Emirates, January 1, 2013–May 9, 2014 (N = 65). Most cases were reported during April 2014.

Most case-patients were male (43/65, 66%) and 20 to 59 years of age (51/65, 78%) (Technical Appendix Table 2). All but 3 expatriate case-patients had been in the UAE for >1 month before diagnosis. However, those 3 had traveled to or lived in the Arabian Peninsula in the month before diagnosis. Forty-two percent of cases were healthcare associated, 34% were epidemiologically unlinked, 19% were household associated, and 6% were related to work or another setting. Eight (12%) cases resulted in death.

### Cluster Identification

We identified 6 clusters. The number of MERS-CoV cases detected by surveillance in 2014 was greater than the number detected in 2013 because of a large cluster that involved both healthcare and household settings in the Eastern Region of UAE during April 2014 (Figure) (*18*). This resulted in 28 epidemiologically-linked MERS-CoV cases. All other clusters in Abu Dhabi—2 healthcare-associated clusters, 1 household-associated cluster, and 2 work-related clusters—involved <5 persons.

### Epidemiologically Unlinked Cases

We identified 22 (34%) epidemiologically unlinked cases in Abu Dhabi (Technical Appendix Table 2); 86% were diagnosed in 2014, and the same percentage occurred in men. Most (59%) epidemiologically unlinked case-patients were 20–59 years of age; median age was 45 years (interquartile range [IQR] 36–66 years). Eight (36%) had traveled outside UAE within 14 days of initial symptoms (3 to Saudi Arabia, 3 to Oman, 1 to Bahrain, and 1 to Thailand). Six (27%) case-patients had documented contact with camels within 14 days before symptom onset or, if asymptomatic, before the date of specimen collection. Eight (36%) case-patients had visited a healthcare setting, such as a dialysis clinic, health clinic, or emergency department, before onset of illness, but these cases were not connected with a known healthcare-associated cluster.

### Disease Severity

A higher proportion of case-patients were male in all 3 disease severity categories (Table 1). The gender disparity appeared to be lower among mildly symptomatic case patients; it was not significantly different for asymptomatic and for severely symptomatic case-patients (p = 0.25; Table 1). Patients who had mild or no symptoms were of similar ages, whereas those patients with severe symptoms were older (median age 37, 42, and 60 years, respectively) (Table 1). Of the 31 healthcare-personnel case-patients, 12 (39%) were asymptomatic. Among case-patients with mild and severe symptoms, 46% and 73%, respectively, had symptoms 1 to 7 days before being hospitalized (Table 1). Duration of patients’ PCR-positivity lengthened as disease severity increased (p = 0.03; Table 2). Of 15 case-patients who had positive test results for >2 weeks, 1 (7%) was asymptomatic.

**Table 1:** Demographic information, symptom duration, and length of positivity by disease severity among MERS-CoV case-patients, Abu Dhabi, United Arab Emirates, January 1, 2013–May 9, 2014*

Characteristic, N = 65	Asymptomatic, n = 23 (35)	With mild symptoms, n = 24 (37)	With severe symptoms, n = 18 (28)	p value†
Sex				0.25
M	16 (70)	13 (54)	14 (78)	
F	7 (30)	11 (46)	4 (22)	
Age, y, median (IQR)	42 (30–54)	37 (30–43)	60 (40–68)	
≤19	0	2 (8)	0	
20–39	10 (44)	13 (54)	4 (22)	
40–59	10 (44)	9 (38)	5 (28)	
≥60	3 (13)	0 (0)	9 (50)	
Healthcare personnel				<0.001
Yes‡	12 (52)	17 (71)	2 (11)	
No	11 (48)	7 (29)	16 (89)	
Symptom duration before hospitalization				<0.001
≥8 d	NA	1 (4)	1 (6)	
4–7 d	NA	6 (25)	10 (56)	
1–3 d	NA	5 (21)	3 (17)	
Same day as admission	NA	9 (38)	2 (11)	
After admission	NA	2 (8)	2 (11)	
Unknown	NA	1 (4)	0	
Length of PCR positivity, d				0.03
<7	15 (65)	12 (50)	12 (67)	
7–14	7 (30)	3 (13)	1 (6)	
>14	1 (4)	9 (38)	5 (28)	

*Values are no. (%) patients except as indicated. Percentages might not sum to 100% due to rounding. IQR, interquartile range; MERS-CoV, Middle East respiratory syndrome coronavirus; NA, not applicable.  †Mantel-Haenszel χ^2^ test. ‡Healthcare personnel were not necessarily part of a healthcare-associated cluster.

**Table 2:** Number of days samples from MERS-CoV case-patients were positive for the virus by rRT-PCR, stratified by disease severity and type of sample, Abu Dhabi, United Arab Emirates, January 1, 2013–May 9, 2014*

Disease severity	No. samples	No. (%) positive LRT samples			No. (%) positive URT samples	
		<14 d	≥14 d		<14 d	≥14 d
Severely symptomatic	67	23 (34)	26 (39)		13 (19)	5 (8)
Mildly symptomatic	148	81 (55)	54 (37)		12 (8)	1 (1)
Asymptomatic	82	60 (73)	5 (6)†		17 (21)	0
Total	297	164 (55)	85 (29)		42 (14)	6 (2)

*Percentages might not sum to 100% due to rounding. LRT, lower respiratory tract; MERS-CoV, Middle East respiratory syndrome coronavirus; rRT-PCR, real-time reverse transcription PCR; URT, upper respiratory tract.  †All 5 lower respiratory samples that were positive for ≥14 d were from 1 asymptomatic case-patient.

### Sample Type and Positivity

Samples from case-patients were tested, at physician’s discretion, while patients were in isolation. Case-patients with severe symptoms had a median of 4 (IQR 2–8, total 103) MERS-CoV rRT-PCR tests, patients with mild symptoms a median of 7 (IQR 3–14, total 214) tests, and patients with no reported symptoms a median of 5 (IQR 4–8, total 147) tests. Most (249/297, 84%) positive results were from lower respiratory tract specimens. Across all disease severity categories, 34% (85/249) of lower respiratory tract samples were positive >14 days after initial positive test for MERS-CoV, whereas 13% (6/48) of upper respiratory tract samples were positive >14 days (p<0.01) (Table 2). Specimens from 6% of case-patients (4/65) remained positive for >3 weeks. These positive specimens included those from 1 case-patient with severe symptoms and from 1 case-patient with mild symptoms whose samples remained positive for 23 days; 1 case-patient with mild symptoms whose samples remained positive for 27 days; and 1 case-patient with mild symptoms whose samples remained positive for 28 days.

Overall, upper respiratory samples were positive less frequently than lower respiratory tract samples (Table 2). In addition, upper respiratory samples were positive less frequently as disease severity lessened (Table 2). Lower respiratory tract samples, however, had a longer duration of PCR positivity than upper respiratory tract samples. For case-patients with severe and mild symptoms, a higher percentage of lower respiratory samples than upper respiratory samples was positive >14 days (for patients with severe cases, 39% vs. 8%; for patients with mild symptoms, 37% vs. 1%) (Table 2).

## Discussion

We describe the public health response and epidemiologic characteristics, clusters, and laboratory results of MERS-CoV case-patients in the Abu Dhabi Emirate of UAE, the country with the third highest number of cases reported to WHO as of July 15, 2015. The Abu Dhabi cases report a relatively low case-fatality rate of 12% compared to the global case-fatality rate of 36% as reported by WHO (*19*); however, surveillance, contact investigations, and reporting methods may vary over time among countries. This descriptive study has several unique characteristics: a large number of cases, including cases from the 3 regions of the Abu Dhabi Emirate; wide range of ages tested; comprehensive contact investigations, including laboratory results; and test results for asymptomatic case-patients. We also report the lengthy duration of viral detection in some asymptomatic case-patients. These findings have useful implications for MERS-CoV management and prevention strategies.

Consistent with previous reports, men were predominantly infected. However, the age group most commonly affected was 20–59 years of age, and 35% of all case-patients were asymptomatic and detected during contact investigations. Those with severe symptoms tended to be >60 years of age, whereas asymptomatic case-patients tended to be younger. This finding agrees with prior case series and cluster analyses in which more severe disease tended to develop in older case-patients (*13*); however, younger persons may have been overrepresented during the contact tracing investigations in our study.

Fever and cough were prominent features of MERS-CoV infection in previous case reports (*6*,*20*), and we found that these symptoms were slightly more frequent among case-patients with confirmed MERS-CoV than among test-negative suspected case-patients. The clinical features of MERS-CoV mimic several other more common illnesses; fever, cough, shortness of breath, and odynophagia were most commonly reported in both case-patients and test-negative suspected case-patients. Therefore, clinicians must continue to maintain a high index of suspicion based on epidemiologic risk factors. More than half of case-patients in our study had contact with another MERS-CoV case-patient, and 20% had some type of animal contact within the previous 14 days. The importance of animal contact is unknown but might be an indicator of camel contact, which has been associated with MERS-CoV infection (*21*).

Most samples from case-patients were taken from the lower respiratory tract, which is believed to be the priority source for specimens for the diagnosis of MERS-CoV (rather than the upper respiratory tract) (*3*,*22*,*23*). In a recent study, Poissy et al. suggested that lower respiratory tract samples are valuable for monitoring MERS-CoV infection (*24*); our results from 65 case-patients with 249 lower respiratory tract specimens further supports this hypothesis. Among all case-patients and disease severity categories, a higher proportion of lower respiratory tract samples were positive >14 days than were upper respiratory tract samples.

Our study identified case-patients who continued to have positive test results for >3 weeks; the longest length of positivity was 28 days in a person with mild symptoms. This finding aligns with results of recent studies that have found case-patients testing positive for MERS-CoV up to 30 days after their first positive test (*24*–*26*). Of the 15 persons in our study who tested positive for >2 weeks, 1 (7%) was asymptomatic and 9 (60%) were mildly symptomatic, which is consistent with a recent case report (*25*). This finding highlights the need to further clarify whether asymptomatic and mildly symptomatic persons play a role in transmitting MERS-CoV to others (*27*).

This study has several imitations. After the merging of 3 independent surveillance systems, some data were incomplete. The case counts increased in April 2014, and HAAD overhauled the surveillance system to meet current epidemiologic needs; this resulted in an inability to compare test-negative suspected case-patients with case-patients after April 17. In addition, characteristics of case-patients may be skewed because most were healthcare personnel from a single hospital cluster. Also, we were unable to correlate timing of reported clinical symptoms with laboratory sample collection in 7 of the symptomatic case-patients, even though multiple data sources were used. Moreover, laboratory specimen collection was not systematic in timing or in type of specimens; laboratory specimens were ordered by physicians at different times during a patient’s hospitalization. Because we reviewed medical charts retrospectively, we were unable to verify whether asymptomatic patients were truly asymptomatic, or if they had undocumented mild symptoms. Although PCR testing provides information regarding viral detection, it does not provide information regarding live virus, and its correlation with virus transmission is unknown. Finally, genetic sequence analysis was able to support the epidemiologic links found in the large healthcare-associated cluster, but not all case-patients had specimens available for genetic sequencing.

In summary, our findings of predominance of male MERS-CoV case-patients, development of more severe disease in older case-patients, and clustering in healthcare settings and household settings are consistent with previous reports (*12*,*20*). This descriptive study also highlights demographic, risk factor, and symptom data related to case-patients tested for MERS-CoV in Abu Dhabi. Our study provides further evidence of a long duration of PCR positivity and the value of using lower respiratory tract samples in monitoring MERS-CoV infection. We also identified asymptomatic and mildly ill MERS-CoV case-patients, which informs practicing clinicians that MERS-CoV causes a wide spectrum of disease. Finally, our study provided a detailed overview of the unique and comprehensive surveillance and response model for MERS-CoV in Abu Dhabi, which included screening symptomatic and asymptomatic case-patient contacts and collecting detailed epidemiologic data on MERS-CoV case-patients. Further studies must investigate characteristics of case-patients, the role of virus detected by PCR in virus transmission, and potential MER-CoV spread from mildly ill or asymptomatic patients to clarify, and ultimately stop, MERS-CoV transmission.

Technical Appendix Characteristics of MERS-CoV case-patients and test-negative suspected patients, Abu Dhabi, United Arab Emirates, January 1, 2013–April 17, 2014.
