# Supplementary material for: Response to Emergence of Middle East Respiratory Syndrome Coronavirus, Abu Dhabi, United Arab Emirates, 2013–2014
Source: Emerg Infect Dis. 2016 Jul;22(7):1162–8. doi: 10.3201/eid2207.160040 (PMC4918155; doi:10.3201/eid2207.160040)
Supplement: Technical Appendix — Characteristics of MERS-CoV case-patients and test-negative suspected patients, Abu Dhabi, United Arab Emirates, January 1, 2013–April 17, 2014. [file 16-0040-Techapp-s1.pdf]

Article DOI: http://dx.doi.org/10.3201/eid2207.160040

Response to Emergence of Middle East Respiratory Syndrome Coronavirus, Abu Dhabi, United Arab Emirates, 2013–2014

Technical Appendix

Technical Appendix Table 1. Characteristics of MERS-CoV case-patients and test-negative suspected patients, Abu Dhabi, United Arab Emirates, January 1, 2013–April 17, 2014 (N = 1,508)*

Characteristic	No. (%) case-patients, n = 41	No. (%) test-negative suspected case-patients, n = 1,467
Year identified		
Unknown	0	134 (9)
2013	11 (27)	669 (46)
2014	30 (73)	664 (45)
Region		
Unknown	2 (5)	995 (68)
Abu Dhabi	13 (32)	301 (21)
Eastern	23 (56)	32 (2)
Western	3 (7)	139 (10)
Sex		
Unknown	0	12 (1)
M	25 (61)	799 (55)
F	16 (39)	656 (45)
Nationality		
Emirati	8 (20)	585 (40)
GCC Country†	2 (5)	260 (18)
Arab (Non-GCC)	7 (17)	94 (6)
Asian	22 (54)	165 (11)
European	0	13 (1)
North American	0	10 (1)
African	2 (5)	23 (2)
Other	0	317 (22)
Age, y		
Unknown	0 (0)	21 (1)
≤19	1 (2)	284 (19)
20–59	31 (76)	889 (61)
≥60	9 (22)	273 (19)
Exposure history		
Any animal	10 (24)	33 (2)
Camel	8 (20)	14 (1)
International travel	6 (15)	222 (15)
Contact with MERS-CoV patient	30 (73)	6 (<1)
Symptoms		
Fever	23 (56)	463 (32)
Cough	18 (44)	461 (31)
Odynophagia	12 (29)	146 (10)
Shortness of breath	10 (24)	185 (13)
Myalgia	10 (24)	46 (3)
Rhinorrhea	8 (20)	92 (6)
Chest pain	5 (12)	50 (3)
Joint pain	4 (10)	28 (2)
Vomiting/nausea	4 (10)	20 (1)
Diarrhea	2 (5)	25 (2)
Abdominal pain	2 (5)	24 (2)
Comorbidities		
Any listed comorbidities	37 (90)	174 (12)
Diabetes	10 (24)	75 (5)

Characteristic	No. (%) case-patients, n = 41	No. (%) test-negative suspected case-patients, n = 1,467
Hypertension	14 (34)	59 (4)
Asthma	3 (7)	40 (3)
Cardiovascular disease‡	2 (5)	11 (1)
Renal disease/failure	7 (17)	6 (<1)
Chronic pulmonary disease	2 (5)	6 (<1)
Obesity	6 (15)	4 (<1)
Type of case		
Healthcare-associated	21 (51)	–
Household	12 (29)	–
Epidemiologically unlinked	8 (20)	–

*MERS-CoV, Middle East respiratory syndrome coronavirus; –, not available. Percentages might not sum to 100% due to rounding.
†GCC countries include Saudi Arabia, United Arab Emirates, Qatar, Bahrain, and Oman.
‡Includes ischemic heart disease, congestive heart failure, and cardiomyopathy.

Technical Appendix Table 2. Characteristics of MERS-CoV case-patients, Abu Dhabi, United Arab Emirates, January 1, 2013–May 9, 2014 (N = 65)*

Characteristic	No. (%) case-patients, N = 65
Year case identified	
2013	11 (17)
2014	54 (83)
Region identified	
Unknown	3 (5)
Abu Dhabi	19 (29)
Eastern	39 (60)
Western	4 (6)
Sex	
M	43 (66)
F	22 (34)
Nationality	
Emirati	17 (26)
GCC country†	4 (6)
Arab (non-GCC)	9 (14)
Asian	32 (49)
African	3 (5)
Age, y	
≤19	2 (3)
20–59	51 (78)
≥60	12 (19)
Exposure history	
Any animal	13 (20)
Camel	8 (12)
International travel	9 (14)
Contact with MERS-CoV patient	40 (62)
Symptom	
Fever	29 (45)
Cough	25 (39)
Odynophagia	19 (29)
Fatigue/malaise	18 (28)
Myalgia	13 (20)
Shortness of breath	11 (17)
Rhinorrhea	11 (17)
Chest pain	6 (9)
Joint pain	5 (8)
Vomiting/nausea	4 (6)
Diarrhea	3 (5)
Abdominal pain	3 (5)
Comorbidities	
Any listed comorbidities	59 (91)
Hypertension	22 (34)
Diabetes	17 (26)
Renal disease/failure	11 (17)
Obesity	7 (11)
Cardiovascular disease‡	4 (6)
Asthma	3 (5)

Characteristic	No. (%) case-patients, N = 65
Type of case	
Healthcare-associated	27 (42)
Epidemiologically unlinked	22 (34)
Household	12 (19)
Work or other setting-related§	4 (6)
Deaths	8 (12)

*MERS-CoV, Middle East respiratory syndrome coronavirus; GCC, Gulf Cooperation Country. Percentages might not sum to 100% due to rounding.
†GCCs include Saudi Arabia, United Arab Emirates, Qatar, Bahrain, and Oman.
‡Includes ischemic heart disease, congestive heart failure, and cardiomyopathy.
§1 case-patient was exposed to another MERS-CoV case-patient at a place of worship.
